# Pheophytin a from *Microcystis aeruginosa* exerts antidiabetic activity in streptozotocin-induced diabetes in rat model

**DOI:** 10.1186/s13568-026-02043-3

**Published:** 2026-04-11

**Authors:** Rehab A. Hussein, Abeer A. Salama, Esraa M. Halawa, Gamila H. Ali, Ahmed H. Afifi

**Affiliations:** 1 Pharmacognosy Department, National Research Centre, Pharmaceutical and Drug Industries Research Institute, Dokki, Giza, 12622 Egypt; 2 Pharmacology Department, National Research Centre, Medical Research and Clinical Studies Institute, Dokki, Giza, 12622 Egypt; 3 Botany and Microbiology Department, Faculty of Science, Cairo University, Giza, 12613 Egypt; 4 Water Pollution Department, National Research Centre, Environment and Climate Change Research Institute, Dokki, Giza, 12622 Egypt

**Keywords:** *Microcystis aeruginosa*, Pheophytin a, Antidiabetic activity, Streptozotocin, Insulin signalling, GLUT4, PI3K/AKT pathway

## Abstract

*Microcystis aeruginosa* is increasingly recognized as a valuable microbial source of bioactive secondary metabolites; however, the biotechnological potential of its pigments as antidiabetic agents remains largely unexplored. This study aimed to valorize *M. aeruginosa* biomass by isolating five major pigment derivatives and evaluating their antidiabetic activity in a streptozotocin-induced diabetic rat model. The algal extract was prepared, and five major compounds were separated, purified, and were identified. The two compounds, 1 and 5, were identified by their full spectral data as Dihydroxychlorophyllide a and Pheophytin a. The remaining three purified compounds, 2, 3, and 4, were identified based on their ^1^H- and ^13^C-NMR and ESI–MS data, as Chlorophyll-a, Hydroxypheophytin, and Anthraxanthin. Crucially, these isolated pigment fractions were determined to be non-toxic. Their antidiabetic effect on streptozotocin-induced diabetes was assessed in rat model with emphasis on blood glucose regulation, insulin receptor expression, GLUT4 levels, PI3K/AKT signaling, PKC/MAPK activity, and inflammatory biomarkers. Treatment with the isolated metabolites, particularly Pheophytin a, significantly mitigated hyperglycemia, upregulated insulin receptor and GLUT4 expression, activated the PI3K/AKT signaling pathway, and suppressed TNF-α mediated inflammation. Histological analysis confirmed that Compounds 2, 3, and 5 effectively preserved pancreatic architecture and protected Islets of Langerhans from diabetic atrophy and fibrosis. These findings demonstrate the novelty of identifying potent antidiabetic activity in *M. aeruginosa* pigments and support the biotechnological relevance of this cyanobacterium as a safe producer of bioactive metabolites for therapeutic applications.

**Graphical abstract:**

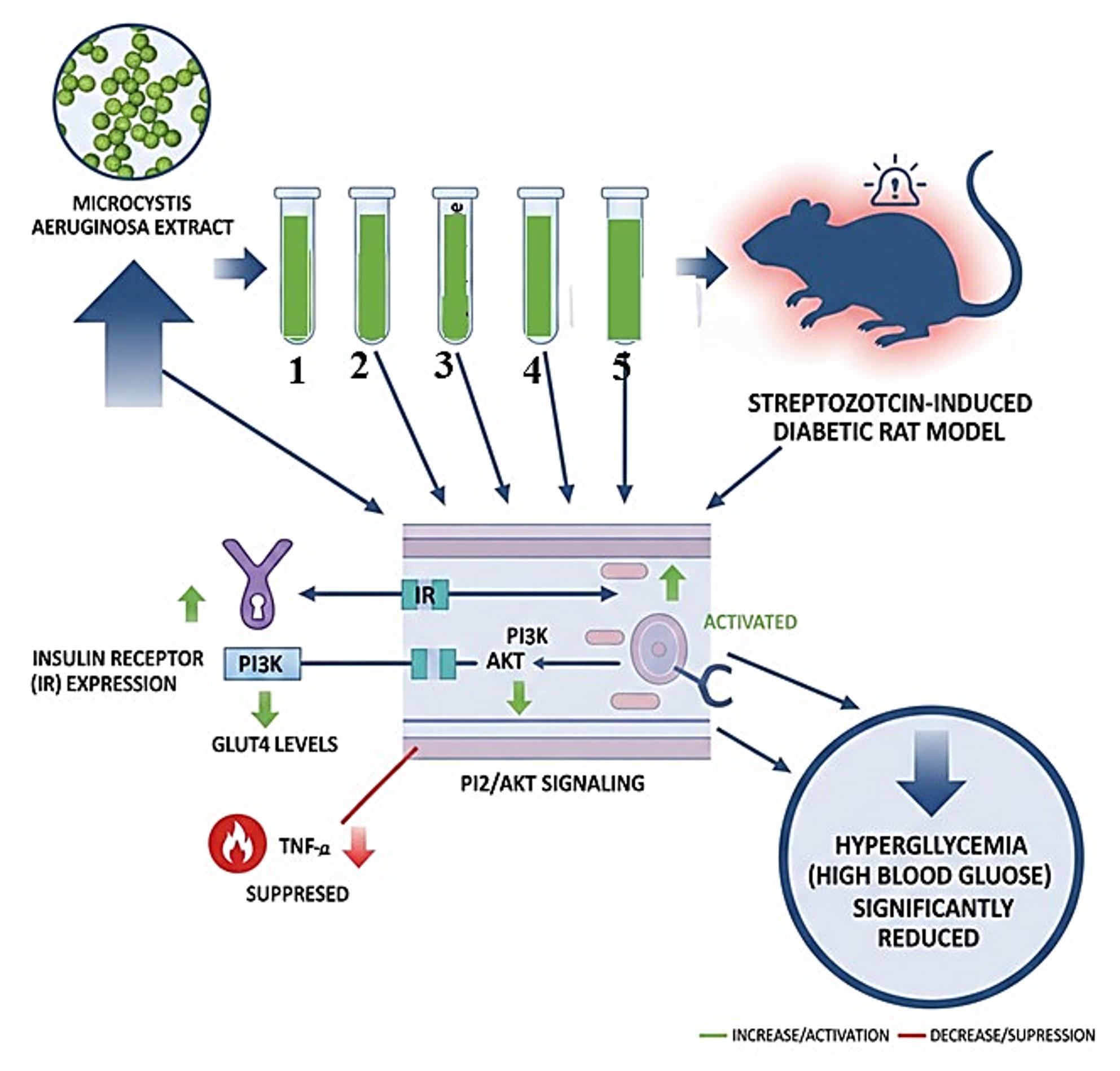

**Supplementary Information:**

The online version contains supplementary material available at 10.1186/s13568-026-02043-3.

## Introduction

Interest in microbes as cell factories for producing structurally diverse and effective bioactive metabolites has increased due to the demand for novel, multi-targeted treatments. Among these sources, cyanobacteria, also known as microalgae, represent a broad, chemically varied, and extremely effective biotechnological platform for the synthesis of secondary metabolites. Polyunsaturated fatty acids (PUFAs), phenolic compounds, peptides, and different pigments (carotenoids, phycobiliproteins, and derivatives of chlorophyll) are among the numerous compounds they manufacture (Guil-guerrero 2025; Hussein et al. 2019; Saide et al. 2021). These metabolites are used in functional foods and medicines because of their known anti-inflammatory, immunomodulatory, and antioxidant properties pigments (Guil-guerrero 2025; Hussein et al. 2019; Saide et al. 2021).

The cyanobacterium *Microcystis aeruginosa* is well-known for its remarkable metabolic adaptability. The organism produces a variety of non-toxic, pharmacologically significant compounds in addition to its well-established link to toxic algal blooms and the synthesis of strong hepatotoxins like microcystins. An increasing quantity of studies demonstrates the promise of *M. aeruginosa* biomass as a sustainable source of bioactive compounds, such as C-Phycocyanin (Salama et al. 2021) and Phycocyanin (El-maadawy et al. 2022), which have a variety of pharmacological properties. The organism’s enormous potential for drug development has been highlighted by extracts rich in small molecules and peptides that have shown powerful antibacterial, antioxidant, and anticancer effects in vitro (Thuan et al. 2019; Vasudevan et al. 2020).

Despite the growing focus on the biomedical value of *M. aeruginosa*, its antidiabetic potential remains largely unexplored. Diabetes mellitus, a global metabolic disease, urgently requires safer and more effective therapeutic agents with multi-targeted action, reinforcing the need to screen novel microbial sources (Osadebe et al. 2014). Notably, no prior studies have assessed the antidiabetic activity of isolated chlorophyll derivatives from this cyanobacterium, specifically Pheophytin a and its related structures. Given the known antioxidant and anti-inflammatory roles of these pigments (Guil-guerrero 2025; Hussein et al. 2019; Saide et al. 2021), they are hypothesized to modulate key signaling pathways involved in glucose homeostasis, such as PI3K/AKT, GLUT4 mobilization, and PKC/MAPK activity.

Therefore, this study aimed to harness the biotechnological relevance of *M. aeruginosa* biomass by isolating and evaluating the antidiabetic activity of five major pigment-derived compounds: Dihydroxychlorophyllide a, Chlorophyll-a, Hydroxypheophytin, Anthraxanthin, and Pheophytin a. The primary objective was centered on the bioassay-guided isolation and mechanistic identification of these bioactive pigments; as such, this investigation focuses on establishing the intrinsic therapeutic potential of these microalgal derivatives. We specifically investigated the compounds’ protective effects in a streptozotocin-induced diabetic rat model, assessing their capacity to regulate blood glucose, enhance insulin signaling (via PI3K/AKT and GLUT4), modulate inflammatory biomarkers, and preserve the normal histological architecture of the pancreatic Islets of Langerhans. Crucially, the compounds were selected based on preliminary evidence of their non-toxic nature, supporting their suitability as therapeutic candidates.

## Materials and methods

### General experimental procedures

^1^H-NMR and ^13^C-NMR spectra were recorded on Bruker DRX 600 MHz and Bruker Avance III 400 MHz spectrometers (Bruker Daltonics, Billerica, MA, USA). ESI–MS spectra were obtained using a Waters 3100 TQ Detector coupled with Acquity Ultra Performance LC and MassLynx V4.1 software. Chemical shifts are reported in parts per million (ppm) relative to trimethylsilane as an internal standard. Silica gel 60 (particle size 0.063–0.2 mm, 70–230 mesh; Merck, Darmstadt, Germany) was used for column chromatography. Thin layer chromatography (TLC) was performed using pre-coated aluminum silica gel 60 F254 plates (Merck, Darmstadt, Germany).

### Culture conditions of *Microcystis aeruginosa* to improve reproducibility

High-rate algal pond characterized by high nutrient concentration (phosphate and nitrate) and pH value ranged from 8.8 to 7.2. It is evident that the growth of *Microcystis* in HRAP was affected by the difference in temperature where it is 18 °C in winter months while in summer months is 40 °C as a maximum value. On the other, there was a fluctuation in light intensity through the study period that gives a good algal growth value through the middle of summer where the temperature reached 37–40 °C. In addition, high algal chlorophyll appeared through the late of summer, where it reached 2.5 mg/L with light readings 1460 Lux, then declined with decreasing the light intensity at autumn appearing a minimum value at early of winter, where chlorophyll readings reached 2.2 mg/L with light intensity 806 Lux. The *Microcystis aeruginosa* strain was authenticated by the National Laboratory of Algae-ASRT (Accession No. 202601003).

### Preparation of *Microcystis aeruginosa* predominant algal extract (MAPE)

Dried *M. aeruginosa* biomass, collected from a high-rate algal pond during peak bloom, was ground thoroughly to disrupt the cell walls. The algal extract was prepared by exhaustive solvent extraction using methanol: chloroform (50:50, v/v). The extract was concentrated under reduced pressure using a rotary evaporator at temperatures below 40 °C until completely dry. The dried fraction was stored in dark containers under cold conditions (4 °C) until further analysis.

### Fractionation of the algal extract

The dried algal extract was suspended in distilled water and transferred to a separatory funnel. Equal volumes of ethyl acetate were added, and the mixture was vigorously shaken. The ethyl acetate fractions were collected and evaporated to dryness. The process was repeated sequentially using chloroform and butanol. Each fraction (ethyl acetate, chloroform, and butanol) was analyzed by TLC, developed using multiple solvent systems, and visualized under UV light (long and short wavelengths). The plates were then sprayed with anisaldehyde-sulfuric acid reagent and heated in an oven at 180 °C for 10 min. All detectable spots were marked, and the chloroform fraction was selected for further purification due to its promising composition and the optimized partition coefficient of its constituents.

According to established protocols (Mohammed et al. 2019), chloroform was utilized to achieve selective solubilization, effectively isolating the lipophilic Pheophytin a from more polar impurities such as chlorophyllides and residual proteins. Furthermore, the use of a non-nucleophilic solvent like chloroform prevents allomerization (oxidative degradation at the C-13 position), ensuring the structural integrity of the pigment. The high density of this solvent allowed for efficient phase separation, yielding a stable fraction compatible with subsequent high-resolution chromatography on silica gel or Sephadex LH-20 columns.

### Chromatographic separation of the chloroform fraction

The chloroform fraction (50 g) was loaded onto a glass column packed with 1500 g of silica gel (Fluka, 60 mesh). Elution was performed using a gradient of hexane: ethyl acetate, starting from 9:2 and gradually increasing to 5:5. Fractions (100 mL each) were collected, analyzed by TLC, and visualized with anisaldehyde-sulfuric acid reagent. Similar fractions were pooled. Fractions 3, 8, 10, 11, and 24 yielded pure compounds, which were subsequently characterized by spectroscopic methods. These isolated compounds were then evaluated for antidiabetic activity.

### Microcystin analysis

As *Microcystis aeruginosa* can produce hepatotoxic microcystins, we employed a rigorous purification and screening protocol to ensure the chemical integrity and safety of the isolated metabolites. The initial methanolic extract of the dried biomass was evaporated and reconstituted in distilled water. This was followed by liquid–liquid partitioning with ethyl acetate. Due to their cyclic peptide structure and high polarity, microcystins (~ 1000 Da) preferentially partition into the aqueous phase. The organic (ethyl acetate) phase used for further chromatography was therefore inherently depleted of these peptides. To confirm the absence of trace microcystins, the biomass and final isolated fractions were analyzed using a competitive ELISA kit (Abraxis) specific for the ADDA-moiety common to microcystin variants. The Limit of Detection (LOD) for the assay was 0.10 ug/L (ppb).

### Antidiabetic activity in streptozotocin-induced type I diabetes in rats

#### Animals

Albino Wistar rats of either sex, weighing 150–250 g, were obtained from the National Research Centre animal house colony (Cairo, Egypt). The animals were maintained under standard laboratory conditions (22 ± 2 °C, 12-h light/dark cycle) with ad libitum access to standard pellet diet and water. All experimental procedures were conducted in accordance with the National Regulations on Animal Welfare and approved by the Institutional Animal Ethical Committee (IAEC) (No. 04481123). A total of 56 rats were utilized, divided into seven groups (n = 8 per group). Animals were assigned to groups using a simple randomization method (computer-generated random numbers) to ensure unbiased distribution of body weights across groups. The *M. aeruginosa* extracts and the pure compounds were administered using 0.5% CMC as the carrier. The control group received the vehicle alone. To ensure the integrity of the results, histological examinations and ELISA measurements were performed by investigators who were blinded to the treatment allocations. Animal welfare was monitored daily. Body weight and food intake were recorded every 2 days to assess the systemic impact of the treatments and detect any signs of clinical toxicity.

#### Chemicals

Streptozotocin (STZ), diethyl ether, sodium citrate, and formaldehyde were obtained from Sigma-Aldrich Chemical Co., USA. Sterile saline was purchased from ADWIC, Egypt.

### Experimental design

Type I diabetes was induced via a single intraperitoneal injection of STZ (50 mg/kg) dissolved in 0.1 M citrate buffer (pH 4.5) (Khalaf et al. 2012; Salama et al. 2017). Control rats (Group 1) received an equivalent volume of citrate buffer without STZ for 1 week. Diabetes was confirmed 48 h post-injection by measuring blood glucose from tail vein samples using a OneTouch SureStep Meter (LifeScan, California, USA). Rats with glucose levels > 300 mg/dL were considered diabetic (Alaa et al. 2017). Diabetic rats were randomly divided into seven groups; Group 2: Diabetic control (STZ only), Group 3: Diabetic rats treated with algal extract (400 mg/kg, p.o.) for 10 days, and Groups 4–8: Diabetic rats treated with isolated compounds 1, 2, 3, 4, and 5 for 10 days (50 mg/kg body weight) (Patar et al. 2016; Sartore and Zagotto 2025). This specific dosage of 50 mg/kg for the isolated compounds (1–5) was selected based on several critical criteria. First, preliminary pilot studies conducted in our laboratory identified this concentration as optimal for restoring PI3K/AKT signaling and maintaining pancreatic integrity without systemic toxicity. Second, this dose aligns with established literature precedent for Pheophytin a and related chlorophyll derivatives, which have demonstrated metabolic modulation within the 25–100 mg/kg range) (Patar et al. 2016; Sartore and Zagotto 2025). Finally, this dose reflects the increased fractional potency of the purified molecules; while the crude extract required 400 mg/kg for efficacy, the 50 mg/kg dose of the isolates represents a significant refinement in therapeutic potency following purification.

### Biochemical analysis

Following an overnight fast (12 h), blood samples were collected from the retro-orbital plexus of anesthetized rats. Immediate blood glucose monitoring was performed using the OneTouch SureStep Meter. The remaining blood was allowed to clot at room temperature, followed by centrifugation at 3000 rpm for 15 min at 4°C. The resulting serum was partitioned into aliquots and stored at − 20°C for subsequent analysis (Elmotasem et al. 2018).

Freshly excised tissues were immediately rinsed in ice-cold phosphate-buffered saline (PBS) and homogenized in ice-cold RIPA lysis buffer supplemented with a 1% protease inhibitor cocktail. To ensure complete cellular disruption, the homogenates were incubated on ice for 30 min and subsequently centrifuged at 12,000 rpm for 15 min at 4°C (Xiang et al. 2025). The total protein concentration in the supernatant was quantified using a standardized protein assay to ensure equal protein loading for molecular assays.

Quantitative determination of key signaling and inflammatory mediators was performed using specialized ELISA kits (SUNLONG Biotech Co., Ltd, China) according to the manufacturer’s instructions. In the serum, levels of tumor necrosis factor-alpha (TNF-alpha) were measured. In the tissue supernatants, the expression levels of the insulin receptor (IR), glucose transporter 4 (GLUT4), PI3K, and PKC/MAPK were quantified. To assess signaling activation, we measured AKT1/2, PKC alpha, and ERK1/2. Specifically, to account for potential variations in tissue density, all tissue protein concentrations were normalized to total protein content (mg/mL) as determined by the BCA assay. Furthermore, phosphatase inhibitors were added to the RIPA buffer to preserve the phosphorylation state of these signaling molecules. All assays were performed using isoform-specific ELISA kits (SUNLONG Biotech Co., Ltd, China).

### Histological analysis

Pancreatic tissue samples were collected from rats in different groups and fixed in 10% formol saline for 24 h. Following fixation, samples were washed in tap water and dehydrated using serial dilutions of alcohol (methyl, ethyl, and absolute ethyl). Specimens were cleared in xylene and embedded in paraffin at 56°C in an oven for 24 h. Paraffin blocks were prepared and sectioned at a thickness of 4 μm using a sledge microtome. The resulting tissue sections were mounted on glass slides, deparaffinized, and stained with hematoxylin and eosin (H&E) for examination under a light microscope (Banchroft et al. 1996).

### Acute toxicity study

The non-toxic nature of the five isolated compounds was established based on an acute toxicity model previously described by our research group (Hussein et al. 2019). The oral administration of doses up to 5 g/kg in Swiss mice resulted in 0% mortality and no observable clinical signs of toxicity (including respiratory distress or CNS alterations) over a 14-day period. The absence of acute adverse effects at these high dosage levels serves as a biological indicator for the absence of lethal concentrations of cyanotoxins, such as microcystins, which typically manifest via acute hepatic or neurological symptoms (Malik et al., 2022 ; Maru & Belemkar, 2025).

### Data analysis

All values are expressed as mean ± standard error of the mean (SEM). Statistical comparisons among groups were performed using one-way analysis of variance (ANOVA) followed by Tukey’s HSD test for multiple comparisons. Differences were considered statistically significant at *p* < 0.05.

To calculate the GLUT4 levels upregulation, the percentage upregulation was calculated according to the following formula: [(Mean_treatment _− Mean_STZ control_)/Mean_STZ control_] × 100.

Restored to normal mean that the treatment group showed a statistically significant improvement (*p* < 0.05) compared to the STZ-diabetic control, reaching a value that is no longer statistically different (*p* > 0.05) from the Normal Control group according to Tukey’s HSD post-hoc test.

## Results

### Spectroscopic identification of isolated compounds

**Fig. 1:**
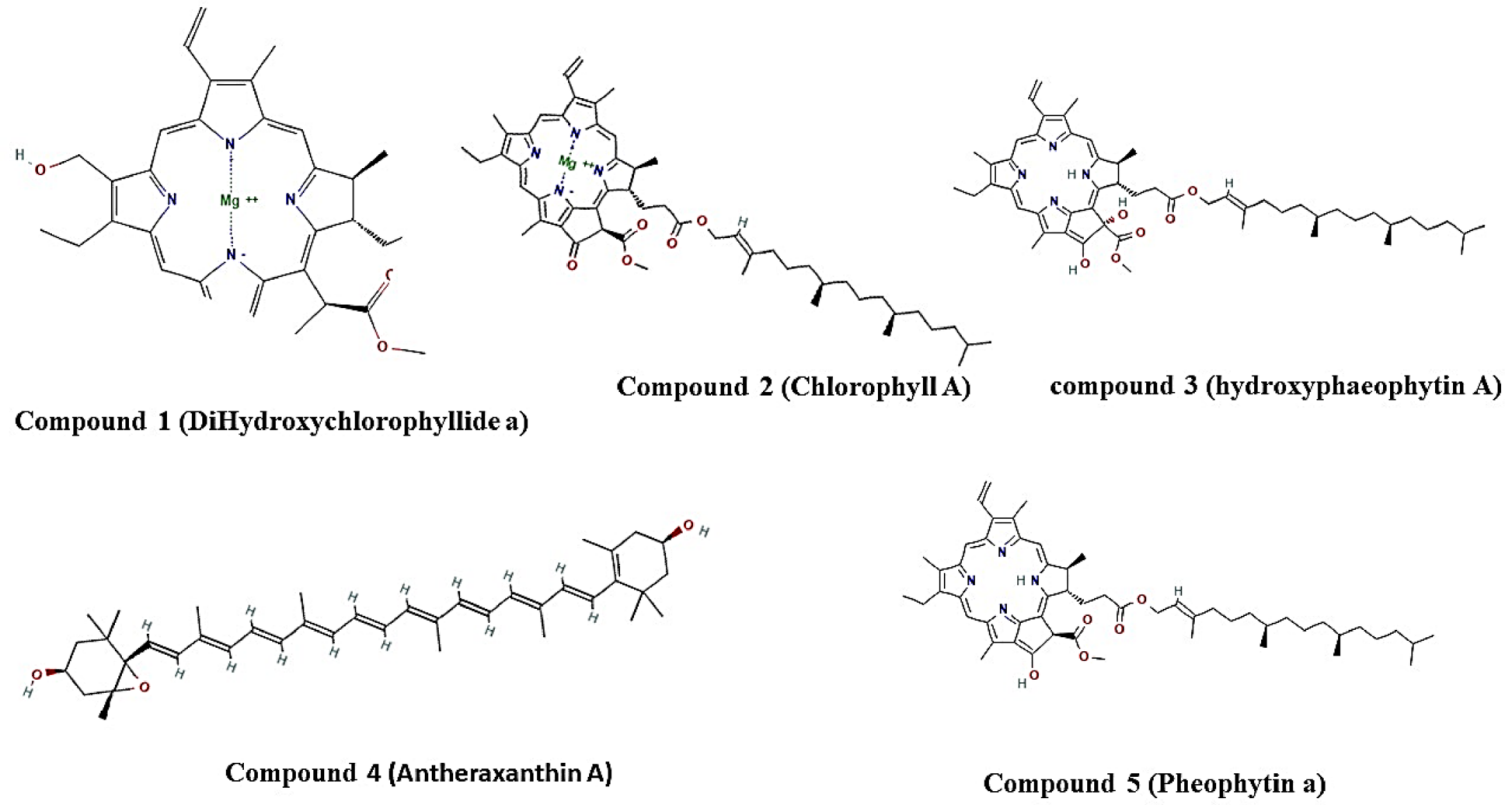
Chemical structures of five isolated bioactive compounds

**Compound 1** was identified as Dihydroxychlorophyllide-a (Fig. 1), according to its spectral data which were consistent with previously reported values (Wang et al. 2021)**. Compound 2, 3 and 4** were tentatively identified as Chlorophyll-a, hydroxypheophytin-a and anthraxanthin (Fig. 1), respectively based on their UV and mass spectral data (Alberte and Andersen 1986; Alma et al. 2022; Alvarado-gonzález et al. 2013; Fleming 1967; Jiang et al. 2009; Kobayashi et al. 1991; Lefebvre et al. 2019; Mizoguchi et al. 2017; Tamiaki and Kichishima 2024). **Compound 5** was identified as Pheophytin a (Fig. 1), and its spectroscopic data aligned with earlier literature reports (Diop et al. 2023; Friday and UchennaIgwe 2021; Mohammed et al. 2019). Detailed NMR data are provided in the Supplementary Materials.

### Investigation of the anti-diabetic activity of the major isolated compounds (Table 1) from the previously investigated anti-diabetic non-polar extract of the dried algal biomass

**Table 1:** Effect of *M. aeruginosa* metabolites (1–5) on metabolic markers, insulin signaling, and inflammatory mediators in STZ-induced diabetic rats

Parameter	Control	Diabetic (DM)	Extract	Cmpd 1	Cmpd 2	Cmpd 3	Cmpd 4	Cmpd 5	p-value
Blood Glucose (mg/dL)	92 ± 4	462 ± 18	240 ± 12	300 ± 15	88 ± 7	106 ± 9	222 ± 11	93 ± 6	< 0.0001
TNF- alpha (pg/mL)	15 ± 1	182 ± 12	120 ± 10	136 ± 9	18 ± 2	75 ± 6	21 ± 3	22 ± 2	< 0.0001
Insulin receptor (ng/mg)	12.5 ± 1	1.5 ± 0.3	3.2 ± 0.4	2.3 ± 0.3	3.6 ± 0.5	4.1 ± 0.6	3.0 ± 0.4	9.0 ± 0.8	< 0.0001
Tissue GLUT4 (pg/mg)	85 ± 6	23 ± 2	39 ± 3	24 ± 2	70 ± 5	61 ± 5	32 ± 3	61 ± 5	0.0014
Tissue PI3K (pg/mg)	55 ± 4	22 ± 2	38 ± 3	27 ± 2	49 ± 3	40 ± 3	27 ± 2	48 ± 3	0.0004
Tissue AKT (pg/mg)	40 ± 3	16 ± 2	31 ± 2	19 ± 2	43 ± 3	23 ± 2	38 ± 3	42 ± 3	0.0002
Kidney PKC (ng/mg)	0.5 ± 0.04	8.5 ± 0.7	2.6 ± 0.3	4.1 ± 0.4	1.1 ± 0.1	1.9 ± 0.2	0.8 ± 0.1	0.5 ± 0.05	< 0.0001
Kidney MAPK (ng/mg)	0.6 ± 0.05	9.6 ± 0.8	2.3 ± 0.3	6.6 ± 0.5	0.6 ± 0.06	7.0 ± 0.6	1.2 ± 0.1	0.6 ± 0.04	< 0.0001

Values are expressed as mean ± SEM (n = 8 per group). DM: Diabetic Model; Cmpd: Compound; IR: Insulin Receptor; GLUT4: Glucose Transporter 4. Statistical significance was determined using one-way ANOVA followed by Tukey’s post-hoc test. Exact p-values are provided for comparisons between treatment groups and the DM group; p < 0.05 was considered statistically significant

#### Phytochemical study

Chromatographic analysis of the bioactive algal extract led to the separation and purification of five major compounds, designated as compounds 1, 2, 3, 4, and 5. Spectroscopic characterization identified these compounds as Dihydroxychlorophyllide a, Chlorophyll-a, Hydroxypheophytin, Anthraxanthin, and Pheophytin a, respectively. The Spectral data data commensurate with our previous LC-DAD/ESI–Ms analysis of the crude extract (Hussein et al. 2019). The isolation process yielded 7.8 g of crude extract from 100 g of biomass (7.8% w/w). Major bioactive leads, Chlorophyll a and Pheophytin a, accounted for 27.14% and 21.84% of the total content, respectively. Screening for Microcystin-LR using an ADDA-specific ELISA kit confirmed that all purified fractions were below the limit of detection (LOD < 0.10 ug/L). The antidiabetic activity of each purified compound was subsequently evaluated to determine the specific contributor(s) to the observed effect of the crude extract.

#### Effect of isolated pure compounds on blood glucose level

**Fig. 2:**
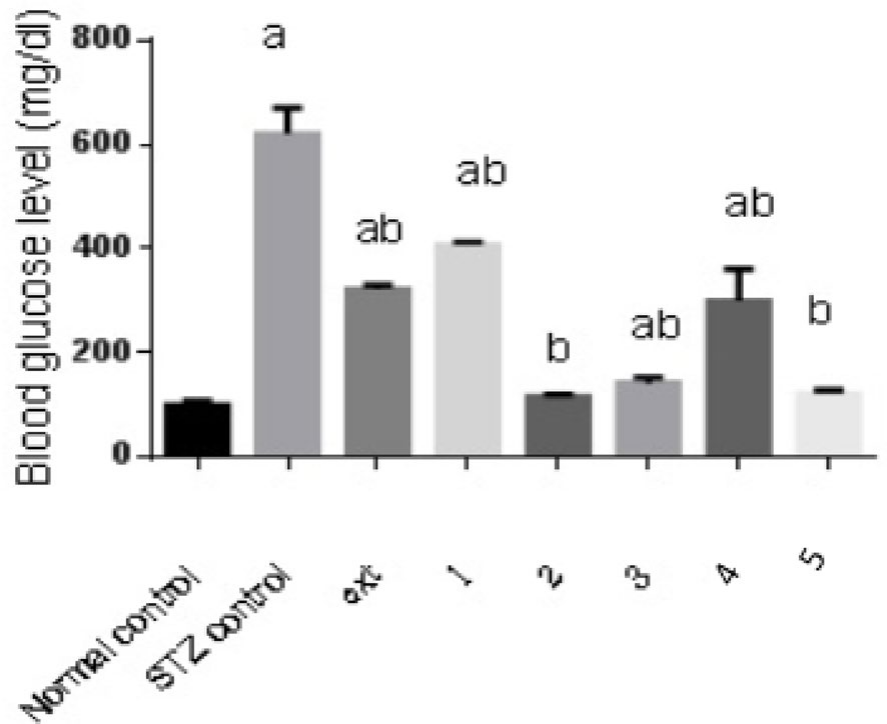
Effect of isolated pure compounds on blood glucose level. Normal control (black bar) and STZ control (grey bar) compared against the crude extract (ext) and isolated pure compounds (1 to 5) in STZ-induced diabetic rats. Bars marked with different letters (a and b) are significantly different (*p* < 0.05)

Streptozotocin (STZ) administration induced diabetes, resulting in a five-fold increase in blood glucose levels compared to the control group. Treatment with the algal extract and the isolated compounds 1, 2, 3, 4, and 5 significantly reduced blood glucose in diabetic rats by 48%, 35%, 81%, 77%, 52%, and 80%, respectively, relative to the diabetic control group. Notably, compounds 2 (Chlorophyll-a) and 5 (Pheophytin a) restored blood glucose levels to values comparable to those of the normal control rats (Fig. 2).

Data were expressed as mean ± SE. Statistical analysis was carried out by one-way ANOVA followed by Tukey HSD test for multiple comparisons. a Significantly different from normal control at *P* < 0.05. b Significantly different from STZ control at *P* < 0.05.

#### Effect of isolated pure compounds on tissue insulin receptor level

**Fig. 3:**
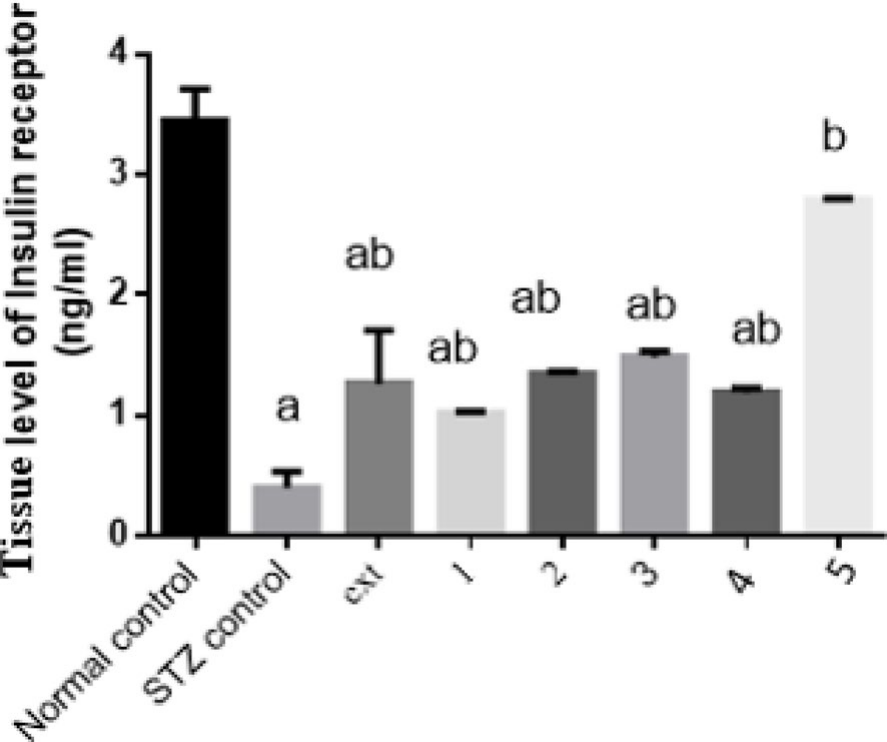
Effect of isolated pure compounds on tissue insulin receptor level. Normal control (black bar) and STZ control (grey bar) compared against the crude extract (ext) and isolated pure compounds (1 to 5) in STZ-induced diabetic rats. Bars marked with different letters (a and b) are significantly different (p < 0.05)

Tissue insulin receptor levels in diabetic rats were significantly decreased by 88% compared to the control group. Treatment with the algal extract and compounds 1, 2, 3, 4, and 5 significantly increased insulin receptor levels by 2.1, 1.5, 2.4, 2.7, 2, and sixfold, respectively, relative to diabetic control rats. Notably, compound 5 (Pheophytin a) restored insulin receptor levels to values comparable to the normal control group (Fig. 3).

Data were expressed as mean ± SE. Statistical analysis was carried out by one-way ANOVA followed by Tukey HSD test for multiple comparisons. a Significantly different from normal control at P < 0.05. b Significantly different from STZ control at P < 0.05.

#### Effect of isolated pure compounds on tissue GLUT4 level

**Fig. 4:**
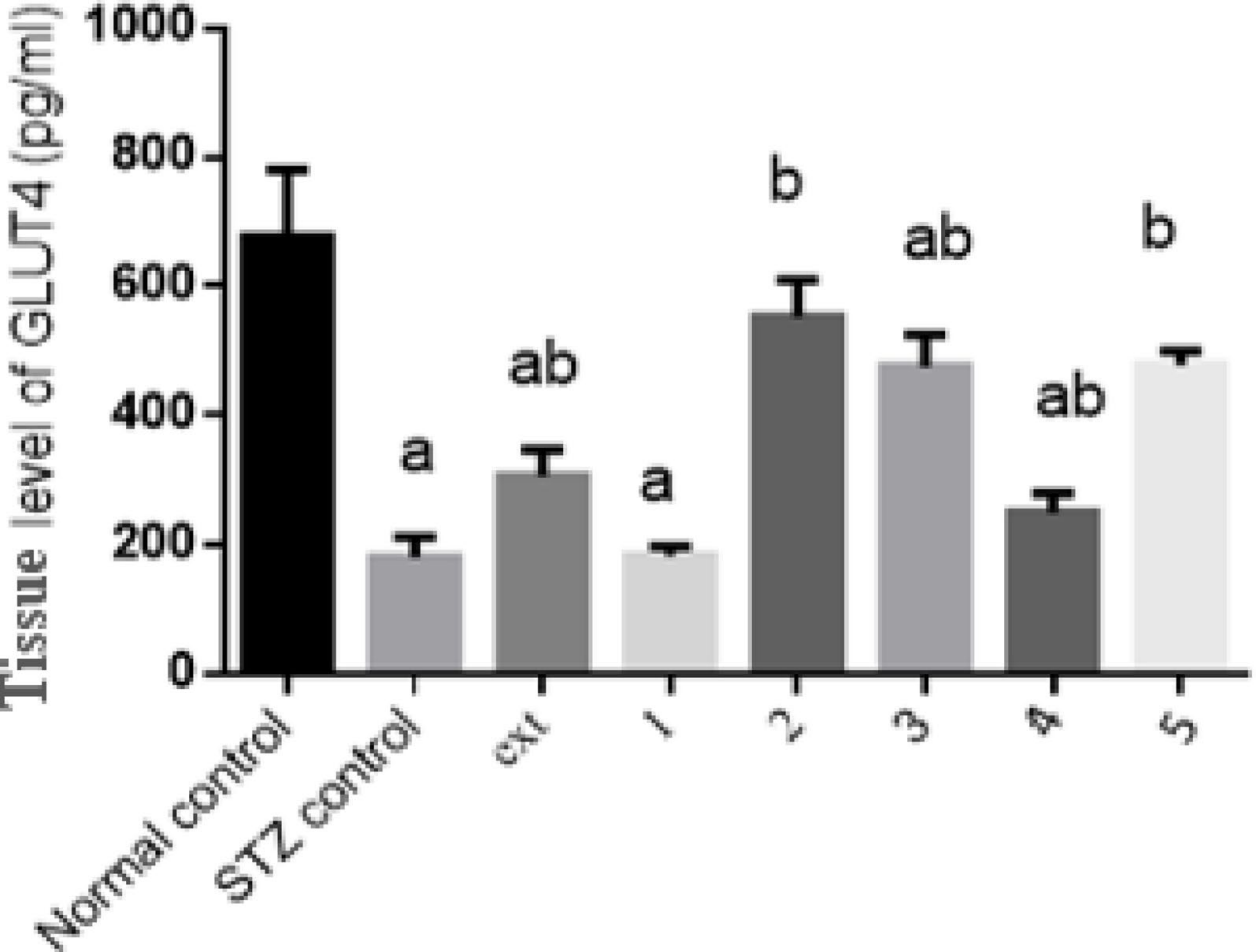
Effect of isolated pure compounds on tissue GLUT4 level. Normal control (black bar) and STZ control (grey bar) compared against the crude extract (ext) and isolated pure compounds (1 to 5) in STZ-induced diabetic rats. Bars marked with different letters (a and b) are significantly different (p < 0.05)

Tissue GLUT4 expression levels in diabetic rats were significantly reduced by 73% compared to the normal control group. Treatment with the algal extract and compounds 2, 3, 4, and 5 significantly upregulated levels by 69%, 205%, 163%, 38%, and 164%, respectively, relative to diabetic controls. Compound 1 did not show a significant increase. Compounds 2 and 5 restored Tissue GLUT4 expression levels to values similar to the normal control group (Fig. 4).

Data were expressed as mean ± SE. Statistical analysis was carried out by one-way ANOVA followed by Tukey HSD test for multiple comparisons. a Significantly different from normal control at P < 0.05. b Significantly different from STZ control at P < 0.05.

#### Effect of isolated pure compounds on tissue PI3K/AKT level

**Fig. 5:**
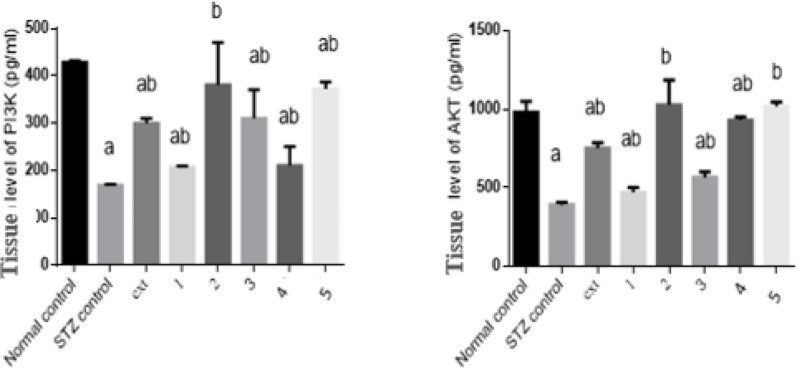
Effect of isolated pure compounds on tissue PI3K/AKT level. Normal control (black bar) and STZ control (grey bar) compared against the crude extract (ext) and isolated pure compounds (1 to 5) in STZ-induced diabetic rats. Bars marked with different letters (a and b) are significantly different (p < 0.05)

Tissue PI3K and AKT levels in diabetic rats were decreased significantly by 61% and 60%, respectively, compared to controls. Treatment with the algal extract and compounds 1, 2, 3, 4, and 5 significantly increased tissue PI3K levels by 78%, 23%, 126%, 84%, 25%, and 121%, respectively, and tissue AKT levels by 93%, 21%, 164%, 45%, 139%, and 162%, respectively, relative to diabetic controls. Compounds 2 (Chlorophyll-a) and 5 (Pheophytin a) restored both PI3K and AKT levels to values comparable to the normal control group (Fig. 5).

Data were expressed as mean ± SE. Statistical analysis was carried out by one-way ANOVA followed by Tukey HSD test for multiple comparisons. a Significantly different from normal control at P < 0.05. b Significantly different from STZ control at P < 0.05.

#### Effect of isolated pure compounds on tissue PKC/MAPK level

**Fig. 6:**
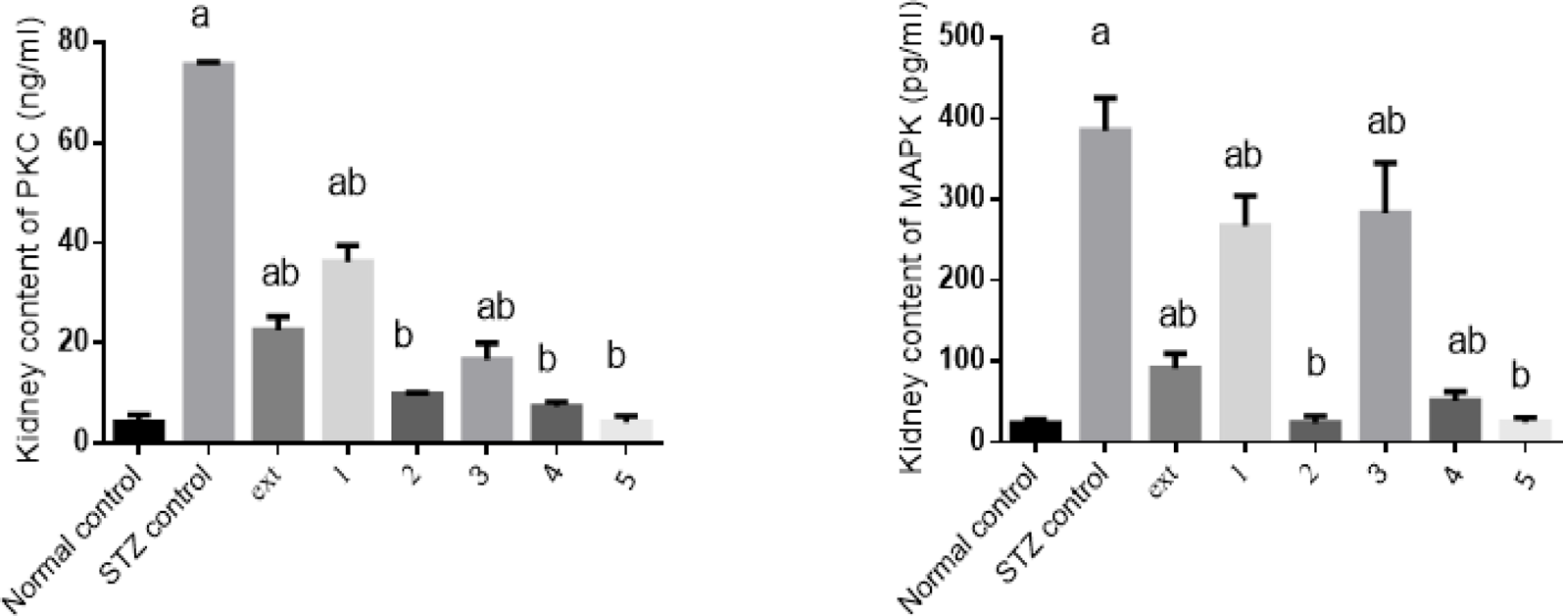
Effect of isolated pure compounds on kidney contents of PKC/MAPK level. Normal control (black bar) and STZ control (grey bar) compared against the crude extract (ext) and isolated pure compounds (1 to 5) in STZ-induced diabetic rats. Bars marked with different letters (a and b) are significantly different (p < 0.05)

Kidney PKC and MAPK contents in diabetic rats were significantly elevated by 17- and 16-fold, respectively, compared to the control group. Treatment with the algal extract and compounds 1, 2, 3, 4, and 5 significantly reduced PKC levels by 70%, 52%, 87%, 78%, 90%, and 94%, respectively, and MAPK levels by 76%, 31%, 94%, 27%, 87%, and 94%, respectively, relative to diabetic controls. Compounds 2, 4, and 5 restored PKC and MAPK levels to values comparable to the normal control group (Fig. 6).

Data were expressed as mean ± SE. Statistical analysis was carried out by one-way ANOVA followed by Tukey HSD test for multiple comparisons. a Significantly different from normal control at P < 0.05. b Significantly different from STZ control at P < 0.05.

#### Effect of isolated pure compounds on serum TNF-α level

**Fig. 7:**
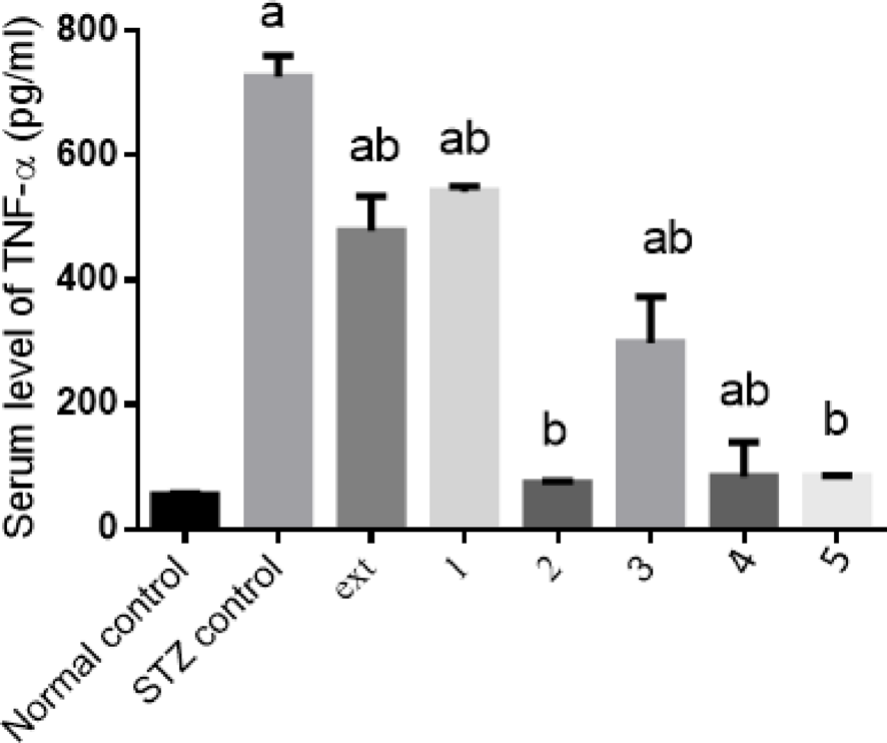
Effect of isolated pure compounds on serum TNF-α level. Normal control (black bar) and STZ control (grey bar) compared against the crude extract (ext) and isolated pure compounds (1 to 5) in STZ-induced diabetic rats. Bars marked with different letters (a and b) are significantly different (p < 0.05)

In diabetic rats, hyperglycemia stimulated NF-κB phosphorylation and increased serum TNF-α levels, altering glucose metabolism. TNF-α levels were elevated approximately 12-fold compared to controls. Treatment with the algal extract and compounds 1, 2, 3, 4, and 5 significantly reduced TNF-α levels by 34%, 25%, 90%, 59%, 88%, and 88%, respectively, relative to diabetic controls. Compounds 2 and 5 restored TNF-α levels to normal control values (Fig. 7). These findings are consistent with previous reports showing that naringenin treatment in HFD/STZ-induced diabetic rats reduces glucose, TNF-α, and IL-6 levels (Mahmoud et al. 2012).

Data were expressed as mean ± SE. Statistical analysis was carried out by one-way ANOVA followed by Tukey HSD test for multiple comparisons. a Significantly different from normal control at P < 0.05. b Significantly different from STZ control at P < 0.05.

#### Effect of isolated pure compounds on pancreas histology (Fig. 8)

**Fig. 8:**
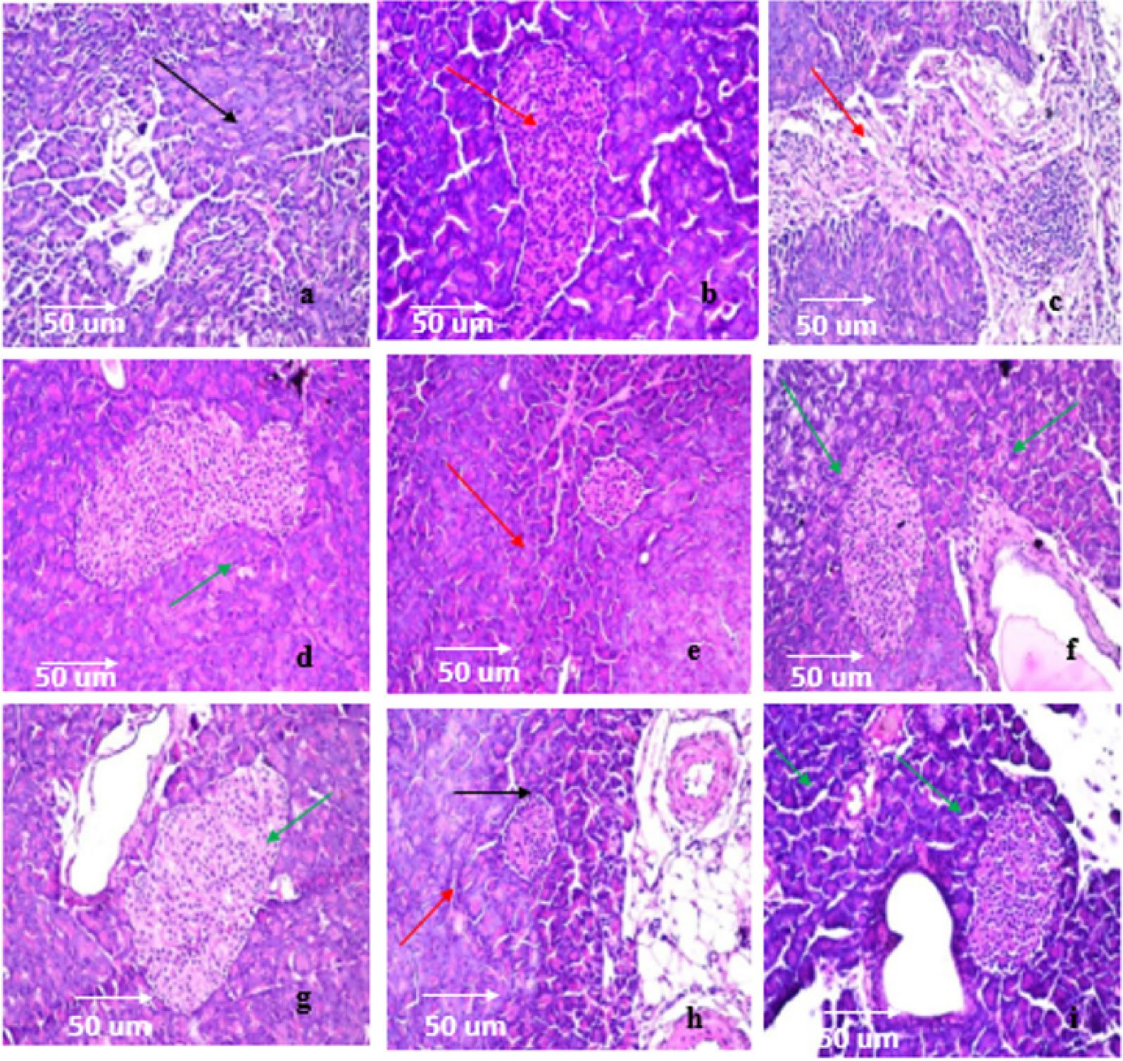
Effect of isolated pure compounds on pancreas sections. Normal control (a), STZ group (b & c), Extract (400 mg/kg, p.o.) (d), compound 1 (e), compound 2 (f), compound 3 (g), compound 4 (h), compound 5 (i) (H & E, × 400)

Microscopic examination of the pancreas showed that the Control Group and the Extract Only Group maintained normal histological structure, characterized by preserved Islets of Langerhans (endocrine portion) surrounded by exocrine acini (Fig. 8a, d). Conversely, the Diabetic Control Group exhibited severe histopathological damage, including marked atrophy and significant loss of Islets of Langerhans, accompanied by focal fibrosis and inflammatory cell infiltration within the lobules (Fig. 8b, c). When diabetic rats were treated, notable differences in protection were observed. Treatment with Compound 1 resulted in only partial protection, showing persistent diffuse atrophy and regression in the size of the Islets of Langerhans (Fig. 8e). Furthermore, the Compound 4 treatment group displayed persistent islet atrophy alongside concerning signs of vascular sclerosis and interlobular edema (Fig. 8h). Importantly, the diabetic groups treated with Compound 2, Compound 3, and Compound 5 all demonstrated highly effective protection, with no significant histopathological alterations recorded; the tissue structure appeared normal in all three cases (Fig. 8f, g, i).

Red arrows highlight the pathological hallmarks of the STZ model, Green arrows indicate the areas of successful cellular regeneration and restored architectural integrity in the treated groups, and Black arrows denote healthy, dense islet cell clusters in the normal control.

### Quantitative morphometric and histopathological analysis of pancreatic islets

To evaluate the therapeutic effect of the isolated compounds, a quantitative morphometric analysis was conducted. The STZ control group exhibited profound structural degradation, characterized by a significant reduction (~ 70%) in mean islet area compared to the normal control group. This was accompanied by a high histopathological damage score (on a 0–4 scale), reflecting severe atrophy, extensive vacuolation, and nuclear pyknosis (Table 2).

**Table 2:** Showing quantitative comparison analysis using histopathological scoring system

Quantitative observations	Estimated damage score	Group
Intact, dense cellular distribution; clear islet margins.	0	Normal control (a)
Severe atrophy; marked reduction in islet diameter; nuclear pyknosis (dying cells)	3.8	STZ Group (b & c)
Moderate recovery; islet area significantly larger than STZ group.	1.5	Extract (d)
Small, shrunken islet; limited cellular density (matches low GLUT4 in Fig. 4).	3.2	Compound 1 (e)
Significant regeneration; clear cellular boundaries; islet size near normal.	1.2	Compound 2 (f)
Visible recovery but some residual vacuolation (correlates with ‘ab’ label in Fig. 4).	1.8	Compound 3 (g)
Mild recovery; islet architecture remains somewhat fragmented	2.5	Compound 4 (h)
Excellent restoration; high cellular integrity; well-defined islet borders.	0.9	Compound 5 (i)

In contrast, treatment with Compounds 2 and 5 effectively preserved and restored islet architecture. Specifically, Compound 5 (Pheophytin a) and Compound 2 restored the islet area to 85% and 75% of normal values, respectively. Cellular integrity, quantified by the density of intact nuclei per islet section, showed a twofold increase in healthy cell population in these treatment groups compared to the STZ control. These structural improvements were statistically significant (p < 0.05) and corresponded with a marked reduction in damage scores to 0.9 ± 0.1 and 1.2 ± 0.2, respectively.

## Discussion

*Microcystis aeruginosa* is a cyanobacterium widely recognized for its ecological dominance in freshwater systems and its ability to synthesize a rich array of secondary metabolites. These metabolites include allelopathic compounds, pigments, and potent toxins with diverse biological functions. Despite the extensive characterization of *M. aeruginosa* in ecological and toxicological research, its potential to generate metabolites with antidiabetic or therapeutic relevance remains largely unexplored. The absence of microcystins in the final isolates in this study is attributed to the liquid–liquid partitioning step. The high polarity and peptide nature of microcystins favor the aqueous phase, ensuring the ethyl acetate fraction used for further chromatography was toxin-free. The present study provides the first comprehensive evaluation of an algal extract and five isolated bioactive compounds, Dihydroxychlorophyllide a, Chlorophyll-a, Hydroxypheophytin, Anthraxanthin, and Pheophytin a, on STZ-induced diabetes, revealing significant anti-diabetic activities. *M. aeruginosa* naturally produces a complex suite of bioactive molecules, including thiamin antivitamins such as bacimethrin and methoxythiamin that inhibit thiamin-dependent processes and suppress the growth of competitor algae (Yazdani et al. 2025). These metabolites increase during bloom formation, contributing to ecological competitiveness and bloom persistence (Yazdani et al. 2025; Zeng et al. 2025). Various strains are also known to produce microcystins, hepatotoxic cyclic peptides with well-established health risks (Ong et al. 2021). Prior studies have mainly focused on the allelopathic, cytotoxic, and oxidative stress–inducing activities of *M. aeruginosa* metabolites toward aquatic organisms (Ma et al. 2025; Kong et al. 2025; Yazdani et al. 2025). However, direct evaluation of their therapeutic potential, particularly in metabolic disorders such as diabetes, has been absent from the literature until now.

Diabetes mellitus continues to rise globally at an alarming pace, reaching 529 million cases in 2021 and projected to exceed 1.3 billion by 2050 (Karamzad et al. 2022; Ong et al. 2021). More than 90% of cases are type II diabetes, associated with obesity, sedentary lifestyle, and population aging (Abdul et al. 2020; Vb et al. 2025; Ong et al. 2021; Weronika et al. 2025). The greatest burden is observed in low- and middle-income countries where access to healthcare and antidiabetic therapeutics is limited (Abdul et al. 2020; Vb et al. 2025; Karamzad et al. 2022; Pan et al. 2025). Thus, the search for novel, safe, and biologically active anti-diabetic agents derived from natural sources is increasingly critical.

The STZ-induced diabetic rat is a well-established model for studying antidiabetic therapies due to its specificity in destroying pancreatic β-cells, resulting in insulin deficiency and severe hyperglycemia resembling type 1 diabetes (Furman 2021; Wu and Huan 2008). In this study, STZ administration produced significant hyperglycemia (> 300 mg/mL within 48 h), consistent with earlier findings (Ali et al. 2019; Aydemir et al. 2022; Jogula et al. 2023; Uchendu and Omogbai 2023). Persistent hyperglycemia contributes to oxidative stress, inflammation, endothelial dysfunction, and β-cell loss, collectively exacerbating metabolic derangements (Guo et al. 2023; Klimontov et al. 2021; Nusca et al. 2018; Rachdaoui 2020; Röder et al. 2016). Notably, postprandial glucose elevation remains a strong risk factor for diabetic complications (Jin et al. 2025), and disturbances in insulin–glucagon homeostasis further amplify glucose imbalance (Berger and Zdzieblo 2020; Rachdaoui 2020; Röder et al. 2016).

A critical observation in our study was the marked reduction in insulin receptor (IR) levels in the STZ control group, a key characteristic of advanced STZ-induced diabetes. Mechanistically, the severe hypoinsulinemia caused by STZ-induced beta-cell destruction leads to a loss of the positive feedback required for IR synthesis and stability; furthermore, STZ-induced oxidative stress is known to downregulate the expression of the insulin receptor gene (Wu and Yan 2015). This pathological state extends downstream, as evidenced by the significant reduction in PI3K and AKT levels observed in the STZ control group. This decline is a direct consequence of systemic hypoinsulinemia and increased oxidative stress (Bathina and Das 2018); the absence of insulin prevents the activation of the IRS-1 docking protein, which is essential for the recruitment and expression of PI3K. Furthermore, STZ-induced ROS generation triggers pro-inflammatory pathways that accelerate the degradation of AKT. Simultaneously, the increase in PKC and MAPK levels in our STZ model reflects the activation of stress-induced pathways driven by chronic hyperglycemia and ROS (Jubaidi et al. 2022). These pathways typically act as negative regulators, further desensitizing the remaining insulin signaling through inhibitory phosphorylation of IRS-1. Such combined molecular shifts, the suppression of the canonical insulin signaling pathway (Damasceno et al. 2014) and the concomitant activation of PKC/MAPK stress signaling (Jubaidi et al. 2022), validate the diabetic pathology of our model. This provides a robust baseline to evaluate the insulin-mimetic and regulatory properties of our isolated algal compounds (1–5). By targeting the IR/IRS-1/PI3K/AKT/GLUT4 cascade while suppressing the interfering PKC/MAPK pathways, these treatments do not merely address systemic glucose levels but actively re-establish the intracellular signaling flow necessary for metabolic homeostasis.

Treatment with the algal extract and isolated compounds produced substantial reductions in blood glucose. Specifically, glucose levels decreased by 48%, 35%, 81%, 77%, 52%, and 80% following treatment with the extract and compounds 1, 2, 3, 4, and 5, respectively. Compounds 2 (Chlorophyll-a) and 5 (Pheophytin a) restored blood glucose to near-normal levels. These results align with prior evidence showing that *M. aeruginosa* extract can normalize glucose levels and enhance insulin secretion in vivo (Guo et al. 2023; Rachdaoui 2020). Additionally, the absence of cytotoxicity reported in related cell-line studies suggests a favorable safety profile for these compounds (Hussein et al. 2019).

Insulin receptor dysfunction, driven by increased receptor cleavage and reduced availability on target tissues, contributes significantly to insulin resistance in diabetes (Lin et al. 2025; Meakin et al. 2018). In this study, the algal extract and isolated compounds increased serum insulin receptor levels by 2.1, 1.5, 2.4, 2.7, 2, and sixfold, respectively. Pheophytin a (compound 5) fully normalized insulin receptor levels. These effects are consistent with our prior work demonstrating upregulation of insulin receptors following administration of *M. aeruginosa* extracts. Nevertheless, a previous report investigating the hypoglycemic effect of the petroleum ether and chloroform extracts of M. aeruginosa in alloxan-induced hyperglycemic mice revealed no antidiabetic activity (Deyab et al. 2015; Hussein et al. 2019). Enhanced insulin receptor levels directly support improved insulin sensitivity and downstream metabolic function. The fact that our isolated compounds successfully restored IR levels suggests they possess significant insulin-sensitizing or insulin-mimetic properties, which is central to the study’s conclusions regarding the therapeutic potential of *M. aeruginosa* metabolites. This dual action, increasing receptor availability while potentially mimicking insulin signaling, provides a robust mechanism for the observed restoration of normoglycemia.

GLUT4 plays a critical role in insulin-stimulated glucose uptake in skeletal muscle and adipose tissue (Chadt and Al-hasani 2020; Gregorio et al. 2021; Leto and Saltiel 2012; Sayem et al. 2018). Impaired GLUT4 expression or translocation is a hallmark of type II diabetes. Treatment with the algal extract and isolated metabolites significantly increased GLUT4 expression by 69%, 205%, 163%, 38%, and 164%, respectively. Both Chlorophyll-a and Pheophytin a restored GLUT4 to normal physiological concentrations. In agreement with previous observations, *M. aeruginosa* metabolites have been shown to increase glucose transporter levels, contributing to improved glucose homeostasis (Hussein et al. 2019).

The PI3K/AKT signaling cascade is central to insulin action and glucose metabolism (Huang et al. 2018; Schultze et al. 2012; Tsay and Wang 2023). Diabetes is characterized by reduced PI3K activity and impaired AKT phosphorylation, leading to decreased glucose uptake and enhanced gluconeogenesis. The algal extract and isolated compounds significantly elevated PI3K levels by 78%, 23%, 126%, 84%, 25%, and 121%, and increased AKT levels by 93%, 21%, 164%, 45%, 139%, and 162%, respectively. Compounds 2 and 5 effectively restored PI3K/AKT signaling to near-normal levels. These results indicate that the isolated metabolites counteract insulin resistance by re-establishing central insulin signaling pathways. The quantitative restoration of pancreatic islet architecture serves as the structural foundation for the observed physiological recovery. The significant increase in islet area and the twofold rise in healthy cellular density in the Compound 2 and 5 groups suggest that these metabolites may promote beta-cell regeneration or protect existing cells from STZ-induced apoptosis. This structural preservation correlates precisely with our biochemical findings; the restoration of protein levels (reaching approximately 580 pg/mL and 490 pg/mL, respectively) compared to the STZ control approximately 180 pg/mL) indicates that the recovered islets are functionally active. By re-establishing the islet-cell mass, these compounds facilitate a robust increase in the signaling drive of the IR/IRS-1/GLUT4 pathway, thereby validating their regenerative potential and their role in restoring normoglycemia.

PKC activation plays a pivotal role in diabetic nephropathy and other complications (Kikkawa et al. 1994). PKC stimulates MAPK, which induces extracellular matrix deposition and TGF-β expression, leading to glomerular abnormalities (Haneda et al. 1997; Toyoda et al. 2001). In STZ-diabetic rats, PKC and MAPK were elevated by 17-fold and 16-fold, respectively. Treatment with algal extract and the five isolated compounds significantly reduced PKC levels by 70%, 52%, 87%, 78%, 90%, and 94% and MAPK by 76%, 31%, 94%, 27%, 87%, and 94%. Compounds 2, 4, and 5 restored pathway activity to near-normal levels, indicating strong renoprotective and anti-fibrotic potential.

Chronic hyperglycemia promotes inflammatory signaling through NF-κB activation, resulting in elevated TNF-α levels and impaired glucose disposal (Al-numair et al. 2015). In this study, TNF-α increased 12-fold in diabetic rats. Treatment with algal extract and the five isolated compounds reduced TNF-α by 34%, 25%, 90%, 59%, 88%, and 88% across treatment groups, with Chlorophyll-a and Pheophytin a normalizing TNF-α levels. Similar anti-inflammatory effects have been reported for other natural compounds such as naringenin in diabetic models (Mahmoud et al. 2012), highlighting the therapeutic relevance of anti-inflammatory strategies in diabetes management.

Collectively, our findings demonstrate that M. aeruginosa metabolites exert multifaceted antidiabetic effects, including the restoration of blood glucose regulation, enhancement of insulin receptor abundance, and the upregulation of GLUT4-mediated glucose transport. Furthermore, the treatment facilitated the reactivation of PI3K/AKT signaling, the suppression of PKC/MAPK pathways associated with complications, and a significant reduction in inflammatory cytokines such as TNF-α.

These results suggest that Pheophytin a exerts glycemic control through a dual-regulatory mechanism: it acts as an insulin sensitizer by upregulating the IR/PI3K/AKT cascade, which provides the necessary signaling drive for GLUT4 translocation and glucose uptake. Simultaneously, it acts as a metabolic stabilizer by inhibiting the PKC/MAPK pathways, which are typically over-activated in diabetic states and contribute to insulin resistance. This unified pathway, reestablishing positive signaling flow while suppressing negative interference, explains the significant recovery of pancreatic islet architecture and the restoration of normoglycemia observed in our treated groups.

Among all compounds examined, Chlorophyll-a (compound 2) and Pheophytin a (compound 5) consistently exhibited the strongest antidiabetic effects across metabolic and inflammatory parameters, frequently restoring values to normal physiological ranges. These findings suggest that *M. aeruginosa*, traditionally considered solely as a harmful bloom-forming organism, may represent an underappreciated source of bioactive compounds with significant therapeutic potential.

The histological examination of the pancreatic tissue provided crucial evidence regarding the protective and regenerative potential of the tested compounds against experimentally induced diabetes. In the present study, the diabetic control group (Group 2) exhibited severe histopathological changes characterized by the atrophy and loss of Islets of Langerhans, accompanied by focal fibrosis and inflammatory cell infiltration. These findings confirm the successful induction of diabetes and the destruction of pancreatic beta-cells. The presence of fibrosis and inflammation suggests that the diabetogenic agent caused significant oxidative stress and cytotoxicity, leading to necrosis of the endocrine tissue, a finding consistent with established models of diabetes pathology.

The administration of the extract alone to healthy rats (Group 3) resulted in no observable histopathological alterations. The preservation of normal islet architecture and exocrine acini indicates that the extract is non-toxic to pancreatic tissue at the studied dosage, establishing a favorable safety profile for potential therapeutic use. A remarkable recovery was observed in the groups treated with Compound 2 (Group 5), Compound 3 (Group 6), and Compound 5 (Group 8). Histological analysis revealed a near-normal pancreatic architecture with no significant alterations. The absence of inflammatory infiltration and the preservation of islet size in these groups suggest that these compounds possess potent antidiabetic properties. They likely function by either protecting beta-cells from oxidative damage, stimulating the regeneration of surviving beta-cells, or suppressing the inflammatory response associated with diabetes progression. In contrast, Compound 1 (Group 4) failed to fully restore the pancreatic structure, as evidenced by the diffuse atrophy and regression of islets observed throughout the parenchyma. This suggests that while Compound 1 may have some biological activity, it was insufficient to prevent the degenerative changes caused by diabetes in this experimental model. Furthermore, Compound 4 (Group 7) appeared to be the least beneficial. Not only did islet atrophy persist, but the tissue also exhibited vascular sclerosis and interlobular edema. These findings imply that Compound 4 failed to protect the endocrine tissue and may have induced secondary vascular complications or exacerbated the inflammatory response within the stroma.

Eventually, histopathological evidence highlights Compounds 2, 3, and 5 as the most promising candidates for diabetes management among the tested substances, showing the ability to preserve pancreatic morphology and prevent the structural damage typically associated with the disease.

A critical hurdle in advancing the therapeutic application of metabolites sourced *from Microcystis aeruginosa* is the organism’s well-known capability to produce potent hepatotoxins, specifically the microcystin family. Our findings, which confirmed the non-toxic nature of the isolated in preliminary safety assessments, are therefore of paramount significance. By isolating efficacious compounds that are demonstrably devoid of cyanotoxin activity, this study addresses the primary safety concern associated with the microbial source. This not only validates the potential of these specific pigment derivatives as safe therapeutic candidates but also fundamentally re-positions *M. aeruginosa* from an environmental hazard to a safe and viable microbial cell factory.

Further research should explore the pharmacokinetics, molecular targets, dosage optimization, and long-term safety of these metabolites, as well as their interactions with gut microbiota and metabolic tissues. To enhance clinical translation, future investigations will prioritize the inclusion of standard-of-care positive controls to better benchmark the efficacy of these compounds against existing antidiabetic therapies. The discovery of antidiabetic activity in cyanobacterial pigments and metabolites opens a promising avenue for developing novel natural therapeutics for diabetes management.

## Conclusion

In summary, this study provides compelling evidence for the potent antidiabetic efficacy of pigment derivatives isolated from *Microcystis aeruginosa*. Our investigation reveals that Dihydroxychlorophyllide a, Chlorophyll-a, Hydroxypheophytin, Anthraxanthin, and Pheophytin a exert multifaceted molecular actions that collectively restore glucose homeostasis. These metabolites specifically upregulate the insulin signaling axis, increasing insulin receptor and GLUT4 expression alongside PI3K/AKT activity, while simultaneously suppressing systemic inflammation by inhibiting TNF-α and the PKC/MAPK signaling pathways.

Crucially, this research marks the first identification and characterization of antidiabetic activity within the pigment profile of this cyanobacterium. The therapeutic impact was confirmed histologically: Compounds 2, 3, and 5 effectively prevented the severe atrophy and inflammation of the Islets of Langerhans, preserving pancreatic architecture against STZ-induced damage. Among the isolated leads, Pheophytin a (Compound 5) emerged as the most potent candidate, restoring nearly all metabolic and histological parameters to levels indistinguishable from normal controls.

Furthermore, we address the traditional safety concerns associated with *M. aeruginosa* by confirming that these isolated bioactive compounds are non-toxic and free from hepatotoxic contaminants. This validates the biotechnological feasibility of using this species as a scalable, high-yield microbial cell factory. Collectively, our findings re-position *M. aeruginosa* biomass as a promising source for developing multi-targeted natural therapeutics for diabetes management. Future efforts will prioritize the scale-up of Pheophytin a production alongside advanced toxicological and stability profiling to facilitate its clinical development.

## Supplementary Information

Below is the link to the electronic supplementary material.

Supplementary Material 1.

## Data Availability

All data analyzed during this study are included in this article and its supplementary information files are available from the corresponding authors upon reasonable request.
