# Supplementary material for: Pheophytin a from Microcystis aeruginosa exerts antidiabetic activity in streptozotocin-induced diabetes in rat model
Source: AMB Express. 2026 Apr 11;16:44. doi: 10.1186/s13568-026-02043-3 (PMC13083692; doi:10.1186/s13568-026-02043-3)
Supplement: Supplementary file 1 — Supplementary Material 1. [file 13568_2026_2043_MOESM1_ESM.docx]

**Supplementary Materials**

**Spectroscopic Analysis and Identification of Compound 1:**

**Fig. S1: ^1^HNMR of compound 1**

**Fig. S2: ^13^CNMR of compound 1**

**Fig. S3: Mass spectum of compound 1**


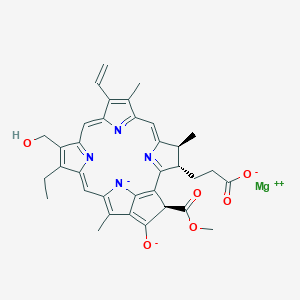


**Fig. S4: Dihydroxychlorophyllide a**

# **Supplementary Tables for Compound 2**

## **Table S1. ¹H NMR Data of Compound 2 (Chlorophyll-a derivative)**

| δ (ppm) | Assignment | Description |
| --- | --- | --- |
| 5.4–5.7 | Vinylic / meso-protons | Protons of conjugated macrocycle |
| 3.3–4.5 | CH₂ near electronegative atoms | Propionate & ester linkage CH₂ |
| 1.8–2.5 | Allylic CH₃ | Methyls adjacent to tetrapyrrole |
| 0.8–1.8 | Aliphatic CH₂, CH₃ | Phytyl chain protons |

## **Table S2. ¹³C NMR Data of Compound 2 (Chlorophyll-a derivative)**

| δ (ppm) | Assignment | Description |
| --- | --- | --- |
| 160–180 | Carbonyl carbons | Ester carbonyls of chlorophyll derivatives |
| >100 | sp² carbons | Porphyrin macrocycle |
| 77.2 | CDCl₃ solvent | Solvent triplet |
| 29–34 | CH₂ | Phytyl chain methylene carbons |
| 22–26 | CH₂ | Additional phytyl chain methylenes |
| 10–20 | CH₃ | Aliphatic methyl carbons |
| 11.1 | CH₃ (C-12a) | Diagnostic methyl of chlorophylls |

## **Table S3. HRMS Data of Compound 2**

| m/z | Ion Type / Fragment | Description |
| --- | --- | --- |
| 647.7090 | Porphyrinic macrocycle ion | Typical for pheophytin/pyro-chlorophyll derivatives |
| 323.3441 | Diagnostic fragment | Common fragment of chlorophyll derivatives |

## **Spectroscopic Analysis and Identification of Compound 3:**

**Fig. S5: ^1^HNMR of compound 3**

**Fig. S6: ^13^CNMR of compound 3**

# **NMR Chemical Shifts of Compound 3 (Hydroxypheophytene-a)**

## **Table S4 ¹H NMR Data of Compound 3**

| Proton Assignment | δ (ppm) | Structural Interpretation |
| --- | --- | --- |
| H-5 (meso) | 10.00 | Chlorin macrocycle meso-H |
| H-10 (meso) | 9.90 | Deshielded meso-H |
| H-20 (meso) | 9.00 | Chlorin macrocycle meso-H |
| Vinyl H-3¹ | 6.29 | Vinylic proton of C-3¹ |
| H-10 (adjacent to OH) | 5.67 | Proton affected by C-10–OH |
| Vinyl/aromatic region | 5.3–7.0 | Chlorin double bonds |
| OCH₃ (C-13³ methyl ester) | 3.84 | Methyl ester group |
| Ring-methyl substituent | 3.67 | Pyrrolic CH₃ group |
| C-17 side-chain CH₂ | 3.90–2.35 | Propionate chain methylenes |
| Allylic/aliphatic CH₃ | 1.83 | Methyl substituent |
| C-8 ethyl CH₃ | 0.90 | Ethyl group of chlorin |

## **Table S4 ¹³C NMR Data of Compound 3**

| Carbon Assignment | δ (ppm) | Interpretation |
| --- | --- | --- |
| C-13¹ carbonyl | 176.31 | Isocyclic ring carbonyl |
| Conjugated macrocycle C=C | 100–160 | Chlorin sp² carbons, vinyl carbon at C-3 |
| CDCl₃ solvent peak | 77.22 | Triplet |
| C-10–OH carbon | 70.59 | Oxygenated carbon confirming hydroxylation |
| Aliphatic CH₃/CH₂ | 14.12–31.93 | Methyl ester, propionate chain, ring methyls |
| C-8 methyl carbon | 14.12 | Methyl group at C-8 |

## **Spectroscopic Analysis and Structural Elucidation of Compound 4:**

**Fig. S7: ^1^HNMR of compound 4**

**Fig. S8: ^13^CNMR of compound 4**

# **NMR Chemical Shifts of Compound 4**

## **Table S5 ¹H NMR** **Data of Compound 4**

| Proton Assignment | δ (ppm) | Multiplicity / Notes | Structural Interpretation |
| --- | --- | --- | --- |
| Vinylic protons (C=CH–) | 5.1–5.4 | - | Partially conjugated polyene system |
| Allylic protons | 2.0–2.8 | - | Protons adjacent to double bonds |
| Aliphatic CH₂ / CH₃ protons | 0.8–1.8 | - | Saturated aliphatic segments of the molecule |

## **Table S6 ¹³C NMR Data** **of Compound 5**

| Carbon Assignment | δ (ppm) | Interpretation |
| --- | --- | --- |
| Olefinic carbons (C=C) | 124–137 | Central polyene chain of Phytoene |
| Methylene carbons (CH₂) | 27.64, 39.79 | Saturated aliphatic segments of the molecule |
| Methyl carbons (CH₃) | <26 | Methyl groups along C₄₀ backbone |

## **Spectroscopic Analysis and Structural Elucidation of Compound 9 (Pheophytin a):**

**Fig. S9: ^1^HNMR of compound 5**

**Fig. S10: ^13^CNMR of compound 5**

**Fig. S11: Mass spectum of compound 5**


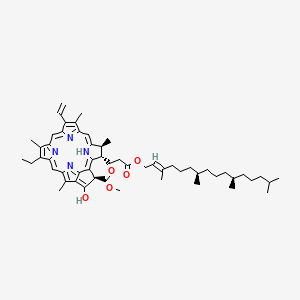


**Fig. S 12: Pheophytin a Structure**

**NMR Chemical Shifts of Compound 5 (Pheophytin a)**

**Table S7 ¹H NMR Data of Compound 5**

| **Proton Assignment** | **δ (ppm)** | **Multiplicity / Notes** | **Structural Interpretation** |
| --- | --- | --- | --- |
| Internal N–H protons | -1.92, -1.72 | - | Porphyrinoid core, confirms demetallation |
| Meso-methine protons | 9.41 (H-5), 8.55 (H-20), 8.18 (H-10) | singlets | Chlorin macrocycle, confirms conjugated tetrapyrrolic system |
| Vinylic protons (C-3 substituent) | 7.80, 6.20 | quartet & multiplet | Vinyl substituent of chlorin ring |
| H-7 | 6.01 | doublet | Partially reduced D-ring |
| H-8 | 5.08 | multiplet | Partially reduced D-ring |
| Methyl ester protons (C-13) | 3.67 | singlet | Phytyl ester side chain |
| Aliphatic CH₂ / CH₃ protons | 0.8–2.3 | - | Phytyl moiety methylene and methyl groups |

**Table S8 ¹³C NMR Data of Compound 5**

| **Carbon Assignment** | **δ (ppm)** | **Interpretation** |
| --- | --- | --- |
| Carbonyl carbons (C-13³ methyl ester, C-17³ phytyl ester) | 173.30, 169.80 | Methyl ester and phytyl ester carbonyls |
| sp² carbons (chlorin macrocycle & vinyl) | 120–160 | Conjugated chlorin macrocycle and vinyl substituent |
| Aliphatic CH₂ / CH₃ carbons (phytyl chain) | 10–70 | Phytyl side chain methylene and methyl carbons |


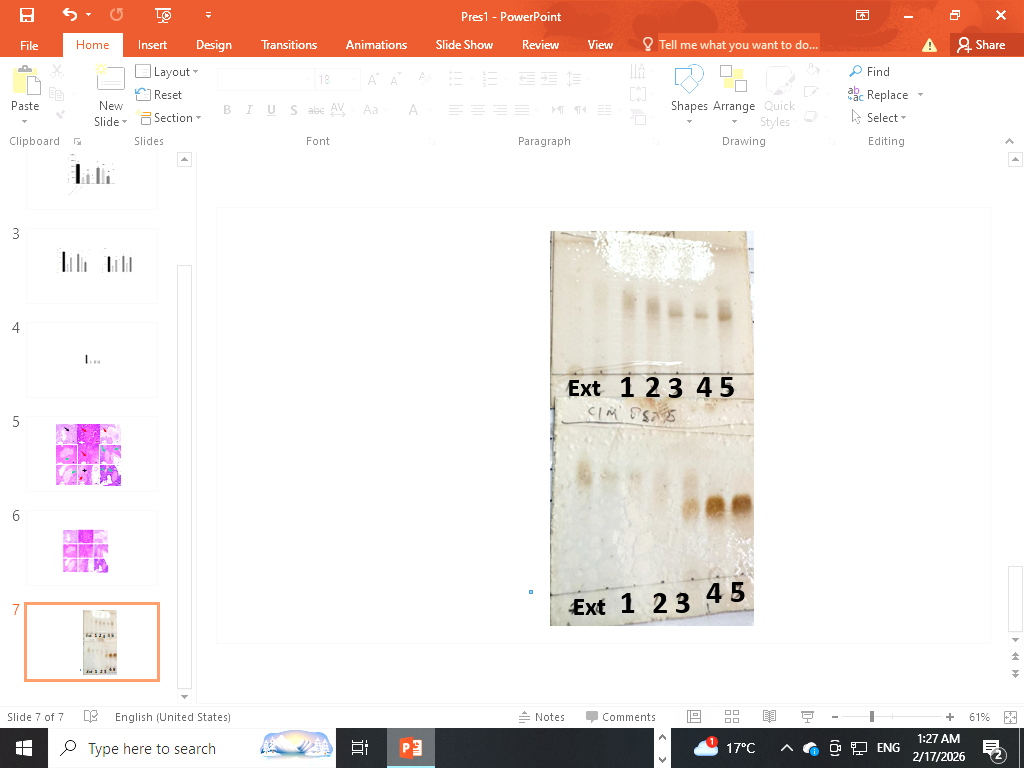


**Fig. S 13: TLC of the isolating compounds**
